# Exploring bone-tumor interactions through 3D *in vitro* models: Implications for primary and metastatic cancers

**DOI:** 10.1016/j.jbo.2025.100698

**Published:** 2025-06-17

**Authors:** Nicolas Cristini, Mohamadreza Tavakoli, Mehdi Sanati, Saber Amin Yavari

**Affiliations:** a Department of Orthopedics, University Medical Center Utrecht, Utrecht, the Netherlands; b Department of Pharmaceutical Nanotechnology, Faculty of Pharmacy, Tehran University of Medical Sciences, Tehran, Iran; c Regenerative Medicine Centre Utrecht, Utrecht University, Utrecht, the Netherlands

**Keywords:** 3D models, *In vitro*, Tumor microenvironment, Bone tumors, Metastatic cancers

## Abstract

Bone tissue serves as a perfect hosting site where metastatic cancer cells of the most prevalent cancer types, such as prostate and breast cancers, as well as the native bone sarcomas, can further proliferate, advancing the disease stage with the consequential decline of the patient’s prognosis. Understanding how the bone niche interacts with tumor cells and the mechanisms leading to drug resistance is a crucial step for enabling the identification of effective cancer therapies. Nevertheless, bone tumor research and the development of new effective anticancer drugs have been hampered for a long time due to the limitations of preclinical models. Traditional 2D cultures and animal models have failed to accurately replicate the human bone cancer microenvironment, driving researchers to develop 3D *in vitro* bone models using tissue-engineered bone constructs and advanced technologies like microfluidics and additive manufacturing. While a complete reproduction of the bone tumor microenvironment (TME), including all relevant cell types, stromal elements, and biophysical cues, remains elusive, targeted inclusion of key components has advanced the physiological relevance of these models. The following review evaluates the biomimetic approaches that have been used to recapitulate the bone TME through 3D *in vitro* models, with particular attention to recent studies aimed at more accurately mimicking the complexity of bone TME, highlighting future directions and the advancements required to overcome present limitations.

## Highlights

• 3D *in vitro* models could replicate the complex bone TME.
• 3D *in vitro* models could overcome the limits of 2D and animal cancer studies.
• Advances in biomaterials and microfluidics improve 3D models’ tumor mimicry.
• Lack of standardization in materials and methods limits clinical translation.
• Future 3D models should integrate patient cells, immunity, and mechanics for realism.

## Graphical abstract

**Figure:**
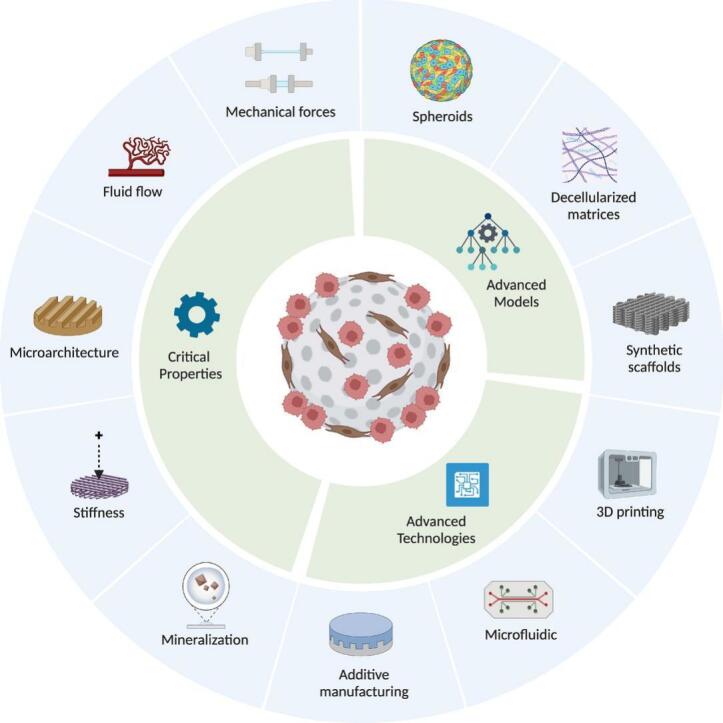

## 1 Introduction

**Fig. 1:**
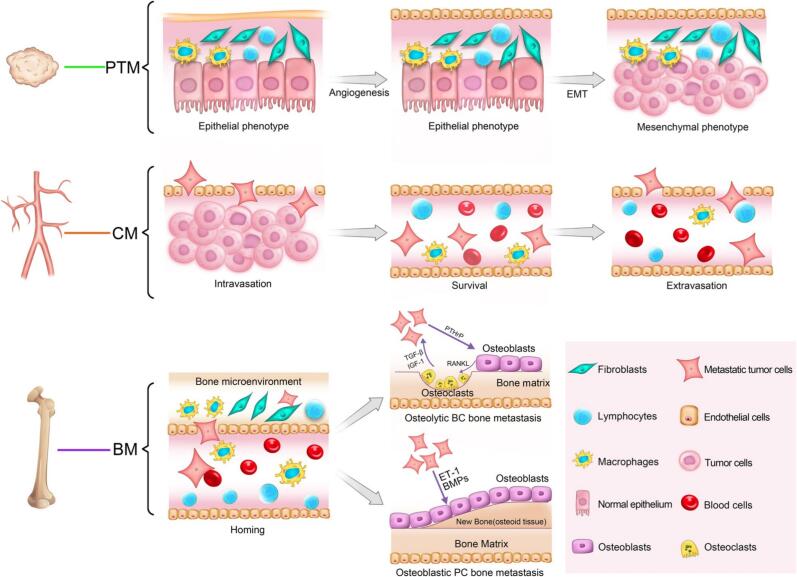
Osteolytic and osteoblastic bone metastasis. Cancer cells initially emerge, proliferate, and acquire invasive traits within the primary tumor microenvironment (PTM). Following detachment, they enter the circulation microenvironment (CM), where they must evade immune surveillance. Upon arrival at the bone, tumor cells engage with the bone microenvironment (BM) and resident stromal and immune cells, facilitating colonization and the formation of osteolytic and osteoblastic metastatic lesions. Reproduced under terms of the CC BY 4.0 license [13]. Copyright 2018, Springer Nature.

Cancer metastasis is a major cause of mortality globally, significantly affecting over 1.5 million patients each year with bone metastases, particularly from lung, breast, and prostate cancers [1,2]. Prostate and breast cancers are the most prevalent malignancies in men and women, respectively, and while they are treatable at early stages, advanced disease frequently results in bone metastases, significantly reducing the five-year survival rate [[3], [4], [5], [6], [7]]. In individuals with osteoblastic metastases, as commonly observed in metastatic prostate cancer, osteoblasts generate new bone of inferior quality, resulting in an increased risk of fractures. This is while osteolytic metastases, frequently associated with breast cancer, can lead to severe pain, pathological fractures due to bone degradation, hypercalcemia, spinal cord compression, and other neurological complications [2]. Fig. 1 demonstrates the dynamic and multistep nature of bone metastasis. In addition to serving as a site for metastatic osteotropic tumors, bone can also develop primary tumors originating from its own microenvironment, e.g., osteosarcoma, chondrosarcoma, and Ewing’s sarcoma. Although rare, these malignancies arise from primitive mesenchymal cells and account for less than 0.2 % of all cancers worldwide [8]. Their complexity and heterogeneity have hindered biological understanding and therapeutic advancements, highlighting the urgent need for comprehensive studies to uncover the molecular mechanisms underlying tumor initiation, progression, and therapy resistance [9].

The increasing prevalence of osteotropic metastatic cancers and the challenges associated with primary bone tumors have driven the advancement of the osteo-oncology field, highlighting the need for a deeper understanding of the molecular mechanisms governing tumor–bone interactions [10]. Traditional 2D cell cultures and *in vivo* models often fall short in mimicking the intricate interactions between tumor cells and bone tissue, limiting their ability to fully capture the biological processes in bone cancers. To address this, the development of 3D *in vitro* culture models that can replicate the unique bone microenvironment and support cancer cell growth has become a key focus in osteo-oncology. These models aim to overcome the limitations of traditional culture platforms, which pose significant challenges for studying tumor–bone interactions [11,12]. This review highlights key advancements in the development of engineered 3D *in vitro* models that replicate tumor–bone interactions, aiming to enhance understanding in this field and provide a foundation for future research focused on overcoming existing limitations.

## 2 Biomimetic 3D cultures: A critical need in cancer research

The high failure rate of potential drugs in clinical trials, largely due to the limited predictive capacity of preclinical models, highlights the need for more advanced testing approaches [14]. Traditional studies using 2D cultures, bone sections, and *in vivo* models remain fundamental but often fail to accurately replicate human tumor complexity and drug responses. Traditional 2D cultures offer several advantages, including cost-effectiveness, speed, and simplicity; however, despite recent advancements such as co-seeding, Transwell membranes, and conditioned culture media, which have improved the study and manipulation of cell populations, these systems still fall short in replicating the intricate complexity of 3D tissue architectures critical for cancer progression and the accurate assessment of therapeutic responses [[15], [16], [17], [18]].

It is demonstrated that the morphology, signaling, and drug response of cancer cells grown in 2D cultures are different from those grown in real *in vivo* conditions [19,20]. For one reason, in 2D cultures, cells are exposed to abundant nutrients and oxygen due to a larger surface area for exchange, reflecting in their proliferation and behavior, thereby failing to simulate diverse cell states of actual tumors [[21], [22], [23]]. Another major limitation of 2D models is the absence of a functional extracellular matrix (ECM) essential for cancer cell signaling, proliferation, migration, and apoptosis [24]. In the tumor microenvironment (TME), the ECM regulates cytokines and signaling pathways and acts as a barrier to drug diffusion, influencing chemotherapy resistance [25]. Consequently, 2D-cultured cancer cells exhibit heightened chemosensitivity due to the lack of ECM protection, potentially leading to misleading drug efficacy assessments. Efforts to improve ECM modeling, such as adsorbing ECM proteins onto glass or plastic, have limitations, as altered protein conformations may distort cellular behavior and responses [26,27]. The limitations and poor predictivity of 2D models highlight the need for alternative systems to improve preclinical outcomes.

Bone sections offer a complex and more real platform for studying cell attachment and cancer-driven bone degradation. For instance, these models have enabled researchers to seed breast cancer cells and evaluate their adhesion, proliferation, and impact on bone resorption [26,28]. Further, nitrogen-containing bisphosphonates have been shown to inhibit MDA-MB-231 breast cancer cell attachment to bone tissue using bone sections, aligning *in vitro* findings with clinical efficacy [29]. However, this approach faces challenges, including the need for fresh bone material, species-specific differences in bone composition, and potential degradation of biological properties during preparation and storage. Additionally, the poor optical properties of native bone can limit microscopy-based assays [30].

*In vivo* studies use either syngeneic or xenograft mouse models. Syngeneic models, recruiting murine cancer cells in immune-competent mice, enable tumor-stroma interaction studies but have limited translational relevance due to species-specific differences in proteins and genes [31]. Xenograft models, utilizing human cancer cells or patient-derived xenografts (PDXs), better preserve human tumor characteristics but require immunocompromised hosts, preventing immune system studies. These models also face challenges such as low engraftment rates, the need for specialized surgical techniques, and ethical concerns regarding high animal usage. Furthermore, differences in pathophysiology and drug metabolism between animal models and humans reduce reproducibility and hinder clinical translation [[32], [33], [34], [35]].

In response, advanced 3D *in vitro* models, as well as patient-derived tumor organoids (PDTOs, miniature 3D tumor cultures grown from patient tumor cells), have been developed to accurately reflect the native architecture and physiological attributes of the TME. These models incorporate physicochemical factors, such as the ECM, stromal cells, and mechanical properties, essential for understanding cancer progression, therapeutic outcomes, and chemoresistance [[36], [37], [38]]. By simulating the dynamic interactions between cells and the ECM found in natural tissues, these models offer a promising approach to better mimic the real *in vivo* environment [39]. Particularly, engineered 3D *in vitro* models integrate native tissue architecture, co-cultures of cancer cells with stromal cells, spatial constraints, and physiological gradients, along with mechanically relevant forces. These advancements not only improve the predictive accuracy of drug screenings but also seek to close the gap between preclinical studies and clinical trials, potentially reducing dependence on animal testing and accelerating the discovery of effective anti-cancer therapies [18,40,41].

Overall, *ex vivo* systems, including engineered 3D models and PDTOs, have advanced tumor modeling and personalized medicine by preserving tumor-specific features and enabling scalable drug screening. Integrating multi-omics with 3D *in vitro* models has also provided new insights into tumor behavior and resistance mechanisms, supporting the development of more precise therapies [42]. However, these models might still lack the full physiological complexity of the *in vivo* TME, which PDX models still best replicate [43]. Despite concerns about proteogenomic divergence of PDXs, they remain the most robust tools for modeling tumor evolution and treatment response [44]. Therefore, while 3D *in vitro* models are advancing rapidly, the consensus remains that they currently complement rather than replace PDXs for robust preclinical prediction.

## 3 Bone homeostasis

Bone homeostasis is a tightly regulated process driven by the complex interplay of various bone cell types. This balance is maintained through continuous bone remodeling, involving two key processes: bone resorption and deposition, which are essential for skeletal integrity and function [45]. Osteoclast precursors, derived from bone marrow monocytes expressing receptor activator of nuclear factor-kappa B (RANK), mature into multinucleated cells capable of degrading the bone ECM [46]. Using enzymes like cathepsin K and acids, they dissolve both organic and inorganic bone components, leading to demineralization [47]. Osteoclastogenesis is governed by interactions between RANK on osteoclast precursors and RANK ligand (RANKL) secreted by osteoblasts and osteoblastic stromal cells. Activated T cells further contribute by secreting RANKL [47]. This process is counterbalanced by osteoprotegerin (OPG), a decoy receptor that inhibits RANKL-mediated bone resorption [48].

Osteoblasts, derived from mesenchymal stem cells (MSCs) in the bone marrow, play critical roles in bone formation. These mononucleated cells secrete collagen and matrix proteins to form the osteoid, which mineralizes through calcium and phosphate deposition. Their proliferation and differentiation are regulated by key growth factors such as PDGF, FGF, and TGF-β [49]. Beyond bone synthesis, osteoblasts contribute to bone homeostasis by modulating resorption through the secretion of RANKL, which promotes osteoclast activation, and OPG, which inhibits osteoclastogenesis [50]. Osteocytes, the most abundant bone cells, are derived from osteoblasts and play a key role in mechanotransduction, adapting bone structure to mechanical stress. They regulate bone remodeling by influencing both osteoclastogenesis and osteoblastogenesis through the secretion of RANKL, OPG, macrophage colony-stimulating factor (M−CSF), and sclerostin [51]. Sclerostin regulates bone mass by inhibiting the Wnt signaling pathway, decreasing under high mechanical load to promote bone formation and increasing under low load to inhibit bone formation [52]. Osteocytes also adjust RANKL production in response to mechanical stress, enhancing bone resorption under low stress and reducing it under high stress to maintain skeletal integrity [53]. Additionally, they act as mechanosensors and contribute to tumor progression by upregulating chemokine (C-C motif) ligand 5 (CCL-5) and matrix metalloproteinases (MMPs) in response to tumor-induced pressure changes [54].

## 4 Key bone properties for biomimetic 3D model design

In recent decades, bone tissue engineering has undergone significant advancements, primarily focusing on the development of tissue-engineered constructs for bone repair [55,56]. These advancements have also facilitated the creation of *in vitro* models that accurately mimic the physicochemical and mechanical properties of natural bone tissue, thereby enhancing regenerative outcomes [57,58].

### 4.1 Mechanical forces

Bone’s high rigidity, in contrast to softer, non-mineralized tissues, necessitates biomaterials with advanced mechanical properties for tissue-engineered constructs [59]. The 3D bone tumor models should replicate the mechanical robustness of natural bone, significantly influencing cancer progression and metastasis. Cancer cells experience various mechanical stresses—tensile, compressive, and shear forces—arising both externally from surrounding tissues and internally from tumor growth [60,61]. These stresses deform blood and lymphatic vessels, limiting oxygen, nutrients, and drug delivery, thereby creating a hypoxic, acidic microenvironment that reduces treatment efficacy [62]. Compressive stresses also enhance cancer cell invasiveness and affect gene expression related to the ECM and vessel remodeling [63]. Chen et al. investigated the mechanical properties of metastatic breast cancer in bone, quantifying the micro-mechanics of tumors and their surrounding bone in a mouse model. Their findings revealed that metastatic tumors had a significantly lower elastic modulus and viscosity compared to *in vitro* cultured cancer cells and *in vivo* subcutaneous tumor explants. This softer nature suggests that metastatic tumors are more deformable, aiding their invasion, adhesion, and spread within the rigid bone environment. Lower viscosity further enhances their adaptability, facilitating movement through the bone marrow network [64]. These mechanical properties may contribute to both therapy resistance and the aggressive nature of bone metastases.

In such cases, 3D *in vitro* models offer a biomimetic approach that not only provides more realistic results but also reduces the reliance on animal models. For example, research on cancer cells seeded onto 3D collagen 1-hyaluronic acid scaffolds and placed in a bioreactor that simulates the mechanical conditions of bone tissue showed that mechanical stimulation enhances runt-related transcription factor 2 (RUNX2) expression and related genes critical for cancer progression in bone. It further identified the extracellular signal-regulated kinase 1/2 (ERK1/2) as a key factor in activating RUNX2 in response to the stimulation, highlighting the ERK1/2-RUNX2 axis as a potential therapeutic target in bone tumors. The research also revealed that mechanical stimulation alters cancer cell drug sensitivity, particularly reducing the effectiveness of RTK inhibitors like sorafenib. Notably, this resistance persisted even after the cells were transferred to standard 2D cultures. Additionally, when patient-derived tumor cells were subjected to similar mechanical conditions in a 3D culture, they maintained their native phenotype better and showed enhanced drug resistance pathways, underscoring the relevance of this model for testing anticancer drugs [65].

### 4.2 Fluid flow and shear stress

As tumors grow, they often develop a hypoxic core, stimulating the production of pro-angiogenic factors such as hypoxia-inducible factor 1-alpha (HIF1α) and VEGF, which promote blood vessel invasion into the TME. These newly formed vessels are typically hyperpermeable and lack a fully developed basement membrane [66,67]. A stable vascular network can be formed using a decellularized 3D bone matrix with endothelial cells and MSCs but fails to replicate fluid dynamics critical for replicating the bone microenvironment [68]. Incorporating advanced techniques like perfusion bioreactors or microfluidic devices can replicate the *in vivo* fluid flow.

Shear stress in tumors, generated by fluid flow within the microenvironment, significantly influences tumor cell behavior and signaling pathways [69]. *In vitro* models incorporated shear stress using perfusion bioreactors demonstrated its impact on cancer cell cycle regulation, gene expression, and chemosensitivity. Bioreactors are essential in 3D models, replicating dynamic forces and enabling long-term studies of bone-tumor interactions, cellular behavior, and intercellular communication [70,71]. Trachtenberg et al. investigated the effects of shear stress on Ewing’s sarcoma cells using poly-propylene fumarate (PPF) scaffolds with varying pore size gradients in a bioreactor. Smaller pores experienced higher shear stress due to increased fluid resistance, while flow perfusion enhanced cell viability and insulin-like growth factor-1 (IGF-1) production. Notably, incorporating larger pores at the top or uniformly medium pores increased IGF-1 production under perfusion conditions [72]. Another example includes incorporating electrospun poly(ε-caprolactone) (PCL) 3D scaffolds within a flow perfusion bioreactor to assess the effects of flow perfusion on Ewing’s sarcoma cell growth and drug sensitivity. Cells were exposed to varying flow rates (0.04, 0.08, and 0.40 mL/min) to mimic mechanical stimulation akin to body fluid movement in the bone microenvironment. Higher flow rates significantly enhanced cell growth and proliferation, as evidenced by increased DNA content. Flow perfusion also promoted more uniform cell distribution compared to static cultures. Although flow conditions did not notably alter the cells' response to doxorubicin, they significantly influenced the cells' resistance to dalotuzumab, a targeted therapy against the IGF-1 receptor. Under flow conditions, tumor cells produced more IGF-1, resulting in increased resistance to dalotuzumab, especially at higher flow rates [73].

**Fig. 2:**
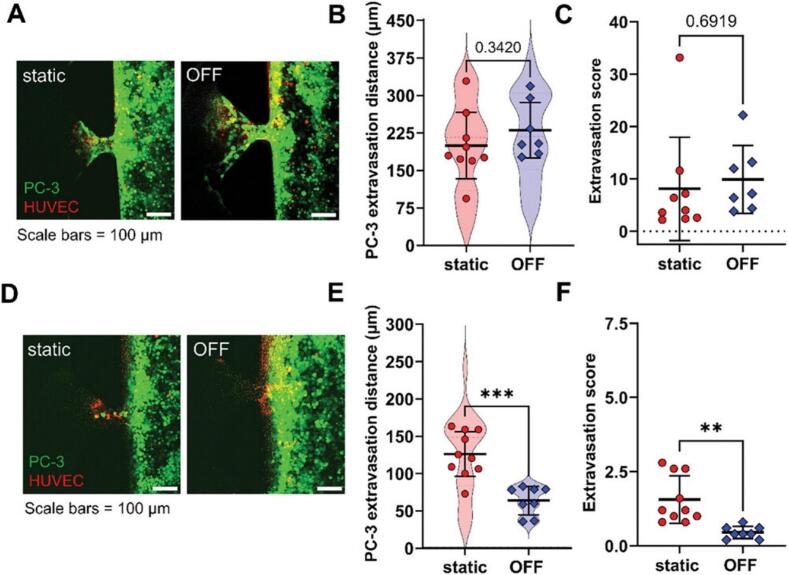
Mechanical loading of MLO-Y4 osteocytes affects PC-3 extravasation. (A) PC-3 cell extravasation toward the osteocyte channel without seeded osteocytes. (B) The superplot of PC-3 cell extravasation distance without osteocytes (C) Extravasation score which quantifies the fraction of affected side channels and the number of extravasated cells in the absence of osteocytes. (D) PC-3 cell extravasation toward MLO-Y4 osteocytes under static and OFF conditions. (E) The superplot of PC-3 cell extravasation distance when MLO-Y4 osteocytes are present (F) Extravasation score in the presence of MLO-Y4 osteocytes. Data presented as mean ± SD. **p < 0.01 and ***p < 0.001 in comparison to the static condition. Reproduced under terms of the CC BY-NC-ND 4.0 license [76]. Copyright 2025, John Wiley and Sons.

Microfluidic devices can support the formation of vascular networks with fluid flow [74]. Researchers employed a microfluidic device to simulate natural interstitial flow, creating a bone perivascular niche-on-a-chip. In this system, bone tissues were exposed to controlled fluid flows, significantly enhancing vascular network formation. Endothelial cells co-cultured with MSCs developed densely interconnected vessel-like structures, in contrast to the sparse networks observed under static conditions. The researchers then introduced MDA-MB-231 breast cancer cells, which formed a triculture environment. The presence of flow resulted in a fourfold reduction in cancer cell growth, suggesting flow-induced inhibition of proliferation. Consistently, sunitinib, a drug targeting dividing cells, was less effective in the dynamic flow-exposed niche compared to static conditions [68]. A study on shear stress in cancer colonization using a 3D microfluidic system also revealed that flow reduced cancer cell extravasation and decreased microvessel permeability, suggesting a strengthened vascular barrier against invasion. Additionally, cancer cells in flow environments migrated further into the surrounding matrix. Flow also influenced endothelial cell morphology, inducing actin filament elongation, junctional stress fiber formation, and alignment in the flow direction, making them more closely resemble *in vivo* microvessel endothelial cells [75]. A recent study explored the role of flow-stimulated osteocytes in early-stage bone metastasis, focusing on prostate cancer-endothelial interactions using a microfluidic tissue model. Findings revealed that osteocytes exposed to fluid flow reduced PC-3 prostate cancer cell adhesion and *trans*-endothelial migration compared to static conditions. Further, murine osteocytes under mechanical loading induced by oscillatory fluid flow influenced the extravasation distance and frequency of PC-3 cells (Fig. 2) [76].

### 4.3 Microarchitecture and surface patterns

In bone tumor engineering, surface properties and interior microstructure of materials are crucial for accurate modeling. Additive manufacturing, such as 3D printing, is key in replicating native bone architecture, enabling the fabrication of bone scaffolds with precise control over microstructure, porosity, and mechanical properties [77,78]. Techniques like extrusion-based manufacturing, inkjet bioprinting, and projection stereolithography allow for the construction of structures essential for realistic bone tumor modeling [79,80]. Other approaches like particulate leaching, freeze-drying, and electrospinning, as well as photolithography have also been used to modify the interior microstructure design and the surface pattern of materials, respectively [[81], [82], [83]].

**Fig. 3:**
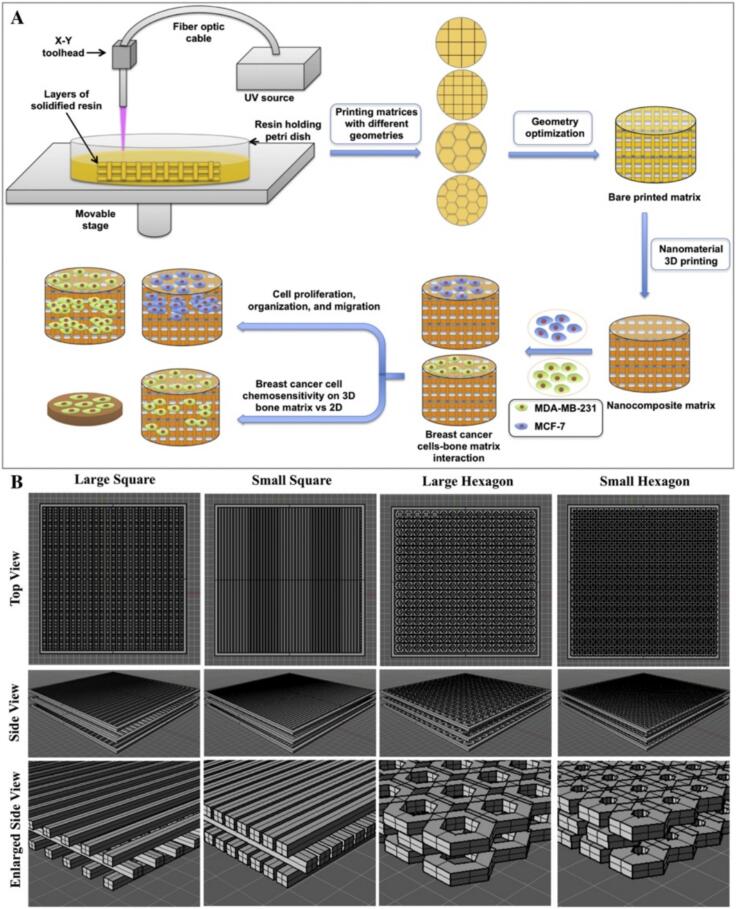
Stereolithography-based 3D printed nanocomposite matrixes for breast cancer bone metastasis. (A) 3D bioprinted bone matrix for investigating breast cancer cell invasion. (B) Top, side, and enlarged computer-aided design illustrations of matrices with large square, small square, large hexagonal, and small hexagonal pore structures. Adapted with permission [84]. Copyright 2016, Elsevier.

Recent advancements in 3D bioprinting and hydrogel-based scaffolds have provided novel insights into the TME and bone matrix interactions. Zhu et al. employed stereolithography-based 3D printing with polyethylene glycol (PEG)/PEG-diacrylate (PEGDA) hydrogel nano-ink to fabricate bone matrix patterns with varying geometric designs (Fig. 3). They observed that reducing channel size (500 µm to 250 µm) increased porosity, while a shift from square to hexagonal shapes decreased porosity. MDA-MB-231 breast cancer cells cultured on these matrices exhibited robust attachment and spreading, with the highest proliferation rate on small square-patterned matrices, highlighting the role of microarchitecture in cellular behavior [84]. Similarly, Jabbari et al. demonstrated that micropatterning in a 3D PEGDA hydrogel significantly influenced tumorsphere growth and cancer stem cell (CSC) marker expression in MDA-MB-231 cells. A 50 µm niche size was identified as optimal for enhancing CSC marker expression, emphasizing the impact of physical constraints on tumor cell plasticity [85].

Extending this concept to osteosarcoma modeling, Negrini et al. developed biomimetic scaffolds by 3D printing polyurethane (PU) matrices and enriching them with bone ECM derived from hMSCs. The scaffold optimization process revealed that a 0.7 mm filament distance provided superior mechanical properties and cytocompatibility. Osteosarcoma SAOS-2 cells exhibited enhanced integration and colonization in ECM-enriched scaffolds compared to controls, underscoring the importance of pre-existing ECM components in modulating tumor–bone interactions [86]. These studies highlight the impact of biomaterial composition and architecture on tumor cell behavior and tissue engineering applications.

### 4.4 Stiffness

**Fig. 4:**
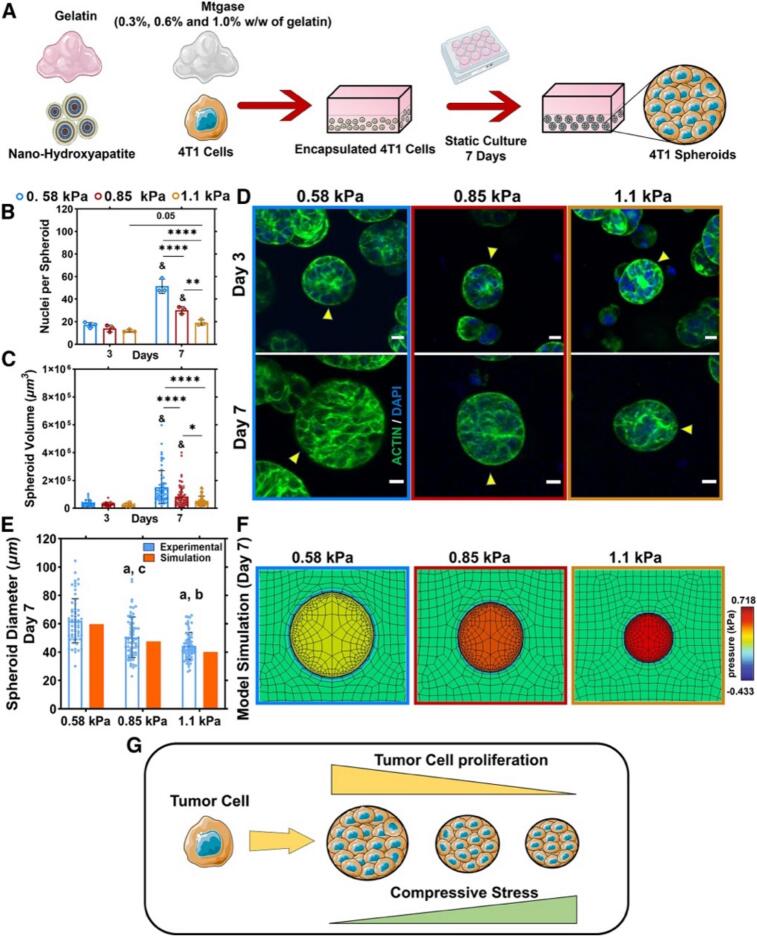
Impact of ECM stiffness on tumor cell proliferation in 3D *in vitro* and computational models of bone metastasis. (A) Encapsulation of 4 T1 cells as single-cell suspensions within gelatin-transglutaminase hydrogels, followed by static culture and spheroid formation. (B) Change in the average number of nuclei per spheroid over time across three hydrogel stiffness levels (0.58, 0.85, and 1.1 kPa). (C) Spheroid volume progression over time under different stiffness conditions. (D) Fluorescent imaging of 4 T1 spheroids (indicated by arrows) within hydrogels, showing actin (green) and nuclei (DAPI, blue) on day 7. (E) Comparison of tumor spheroid formation in hydrogels on day 7 with computational model simulations of tumor growth at the same stiffness levels. (F) Computational model illustrating spheroid expansion within hydrogels (green background) and the corresponding stress distribution. (G) Correlation between tumor spheroid growth and hydrogel stiffness. Data are presented as mean ± SEM. & (p ≤ 0.05) vs. day 3; a, b, c (p ≤ 0.05) vs. 0.58, 0.85, and 1.1 kPa, respectively; *p ≤ 0.05, **p ≤ 0.01, ***p ≤ 0.001, ****p ≤ 0.0001. Reproduced under terms of the CC BY-NC 4.0 license [90]. Copyright 2024, Cell Press. (For interpretation of the references to colour in this figure legend, the reader is referred to the web version of this article.)

The variability in the mechanical properties of trabecular bone, with a wide range of stiffness and elasticity, highlights the complexity of bone tissue [87]. Notably, the mechanical properties of the ECM regulate tumor growth and CSC behavior. Charoen et al. demonstrated that collagen gel density significantly influences spheroid growth. U2OS spheroids exhibited optimal expansion in 4 mg/mL collagen gels, favoring a stiffer environment, whereas MDA-MB-231 spheroids thrived in softer 2 mg/mL gels [88]. Bassi et al. investigated CSC behavior in two 3D bone-mimicking scaffolds of varying mechanical properties: a low-stiffness hybrid scaffold composed of Mg-doped hydroxyapatite with collagen fibers and a high-stiffness porous hydroxyapatite scaffold. CSC-enriched sarcospheres from MG63 and SAOS-2 cell lines maintained their spheroidal morphology and CSC marker expression in both scaffolds. Notably, SAOS-2 sarcospheres exhibited enhanced stemness gene expression within the stiffer scaffold [89]. Investigating the impact of matrix stiffness on growth and CSC marker expression across multiple cancer types using a PEGDA hydrogel system revealed that tumors from different tissue origins exhibit distinct biomechanical preferences. Breast cancer cells favored a softer (5 kPa) matrix, colorectal and gastric cancer cells required an intermediate (25 kPa) stiffness, while osteosarcoma cells needed the stiffest (50 kPa) environment [85]. Consistently, Kuma et al. developed a mechanobiological model of bone metastasis, showing that mechanical stimulation suppresses the pro-osteolytic effects of breast cancer cells. They cultured 4 T1 breast cancer cells in gelatin-transglutaminase hydrogels of different stiffness levels and observed spheroid formation over seven days. As hydrogel stiffness increased, spheroid size and cell count decreased (Fig. 4). To further analyze stress-dependent growth, they created a computational model, which revealed that higher circumferential and radial stresses in stiffer environments slow cell proliferation, indicating the role of mechanosensitivity in tumor growth regulation [90]. These findings underscore the necessity of tailoring ECM stiffness when designing *in vitro* cancer models, as well as the tissue-specific influence of matrix rigidity on tumor progression.

### 4.5 Mineralization

The bone ECM is a complex network of proteins and mineralized substances that supports bone structure and mechanics. Hydroxyapatite, making up 50–70 % of bone, is valued for its biocompatibility and osteoconductivity. When combined with polymers, it enhances osteoinduction and flexibility, aiding in biomaterial development, especially for 3D bone cancer modeling [91,92]. Several investigations explored the effects of hydroxyapatite nanoparticles on bone-mimicking scaffolds and breast cancer cell behavior. Pathi et al. examined hydroxyapatite nanoparticles with different crystallinity and sizes in poly(lactide-co-glycolide) (PLGA) scaffolds, finding that smaller, less crystalline hydroxyapatite enhanced cancer cell adhesion and proliferation, while larger, more crystalline hydroxyapatite increased interleukin-8 (IL-8) expression, promoting osteolysis [93]. Zhu et al. studied hydroxyapatite/chitosan composites, showing that hydroxyapatite size, crystallinity, and concentration significantly influence breast cancer cell adhesion. Amorphous hydroxyapatite reduced, while crystalline hydroxyapatite (nano- and micro-sized) enhanced adhesion. Additionally, lower hydroxyapatite concentrations improved adhesion by preventing agglomeration [94]. Although both studies emphasize the role of hydroxyapatite properties in cancer cell interactions within bone scaffolds, more investigations are required to clarify further the impact of incorporating a mineral phase.

## 5 Approaches for 3D modeling of bone tumors

Natural biomaterials, such as proteins, polysaccharides, and decellularized bone tissues, are used in *in vitro* 3D bone tumor models for their biocompatibility and ability to mimic native cell-matrix interactions [12,95]. While synthetic materials offer greater control over mechanical properties, natural biomaterials excel in regulating cellular behavior [96,97]. Common synthetic materials, including PCL, PLGA, PEG, and polyacrylamide (PAM), are widely used in bone tissue engineering, often combined with hydroxyapatite or other bioceramics to enhance bone-like properties [84,93,98]. Biomaterials can be modified with specific ligands like RGD, IKVAV, and GFOGER to study cancer cell behavior in response to ECM proteins [99]. Biomaterials also help investigate the ECM's role in cancer progression, particularly how cancer cells degrade their surroundings using MMPs [100]. For example, hydrogels, such as MMP-degradable hyaluronic acid-based hydrogels, enable research on tumor invasion, showing that metastatic cells invade deeper in degradable environments [101]. This section discusses various approaches, techniques, and biomaterials for 3D modeling of bone tumors.

### 5.1 Multicellular tumor spheroids

**Fig. 5:**
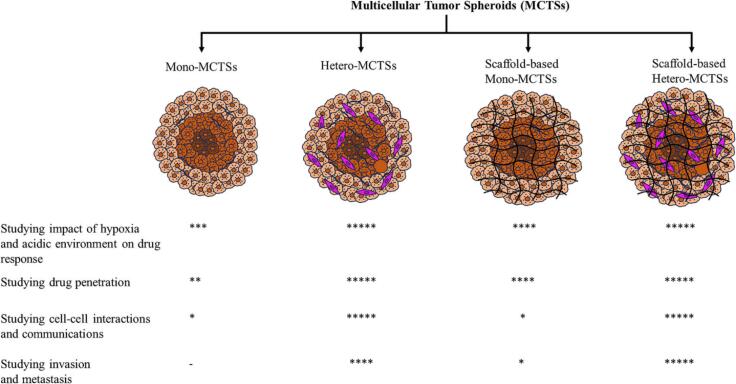
Various approaches for MCTS generation for cancer biology and drug screening investigations. Mono-MCTSs are monocellular tumor spheroids, also called homospheroids. Hetero-MCTSs are co-culture tumor spheroids, also called heterospheroids. Scaffold-based MCTSs include scaffold-based Mono- or Hetro-MCTSs. Adapted with permission [102]. Copyright 2024, Elsevier.

Multicellular tumor spheroids (MCTSs), or tumorspheres, were among the first 3D cell culture methods. They form spontaneously through cell–cell interactions, replicating key *in vivo* tumor characteristics such as cellular heterogeneity, metabolic gradients, and gene expression patterns [102,103]. In larger spheroids, oxygen, pH, and nutrient gradients create distinct growth zones, including a hypoxic core. These features, along with restricted drug diffusion, make MTS more physiologically relevant than traditional 2D cultures [104,105]. Various methods have been recruited for MCTS generation, each with advantages and limitations. The forced floating and hanging drop methods, along with innovations like superhydrophobic chips, demonstrate the adaptability of MCTSs for high-throughput drug screening [[106], [107], [108], [109], [110]]. However, a key limitation of MTS is their inability to fully replicate the tumor microenvironment, particularly the role of the ECM in tumor progression and therapy resistance. To address this, scaffold-based approaches incorporating natural or synthetic hydrogels have been developed, offering a more physiologically relevant model by supporting tumor organization and invasive behavior (Fig. 5) [102,111].

Studies further refine these models to enhance reproducibility and clinical relevance. Charoen et al. demonstrated a strong correlation between cell seeding density and spheroid size using U2OS and MDA-MB-231 cancer cell lines. Their findings highlighted a pattern of live cells on the spheroid's periphery and dead cells at the core, mirroring metabolic gradients of actual tumors and reinforcing the biological relevance of 3D models [88]. Jabbari et al. studied the effect of initial cell seeding density on tumorsphere formation using MDA231 breast cancer cells encapsulated in PEGDA hydrogels. At low densities, cells remained singular with no significant growth or CSC marker expression. High densities increased cell numbers but did not promote tumorsphere formation. However, at moderate densities, tumorspheres formed successfully, showing increased CSC marker expression, indicating an optimal density range for tumorsphere development [85]. Another investigation developed a hydrogel-based 3D *in vitro* model by embedding MG-63 spheroids into ECM-mimetic scaffolds comprised of gelatin methacryloyl (GelMA) or Matrigel. Using 30,000 cells, compact spheroids were formed in the hydrogels, which exhibited greater invasion and drug resistance than dispersed cells. The study also demonstrated increased metabolic activity in spheroids over time, validating the model's effectiveness in simulating tumor growth and drug response [112].

### 5.2 Cell-derived matrices

Cell cultures like osteosarcoma cells, pre-osteoblasts, and primary osteoblasts help develop ECMs that mimic the bone environment [113,114]. These matrices are useful for studying cancer-bone interactions by replicating bone development and cancer progression [[115], [116], [117]]. However, scaling them for broader applications is challenging due to complex manufacturing and the need for greater stiffness to match bone mechanics. Krishnan et al. demonstrated that a 3D cell-derived matrix effectively replicates the bone microenvironment within a bioreactor system for osteoblastic tissue cultivation. Composed primarily of ECM proteins secreted by maturing osteoblasts, this matrix supported the proliferation and differentiation of MC3T3-E1-derived osteoblasts into a multilayered structure resembling *in vivo* bone tissue. The 3D bioreactor model enabled cancer cell infiltration through osteoblastic layers and cancer-induced morphological changes in osteoblasts. Notably, the study highlighted that the osteoblast maturation stage significantly impacts metastatic breast cancer cell colonization, with less differentiated osteoblasts promoting greater cancer cell proliferation and adhesion [118].

Taubenberger et al. further explored metastatic breast cancer interactions using a human primary osteoblast-derived matrix (hOBM), which closely mimics native bone ECM. The hOBM exhibited a complex fibrillar structure, high mineral content, and the presence of essential bone proteins. This matrix not only supported breast cancer cell adhesion and proliferation but also influenced key metastatic behaviors. Metastatic cell lines such as MDA-MB-231 and SUM1315 exhibited persistent and directed migration and invasion on hOBM, indicating its relevance for studying cancer metastasis and assessing anti-migration therapies [119]. Another investigation developed an *in vitro* model utilizing osteoblast-derived matrices (OBM) to investigate prostate cancer interactions within the bone microenvironment. Following decellularization, the OBM retained its fibrillar ECM structure, closely replicating the mineral and protein composition of natural bone, including calcium phosphate and key bone-specific proteins such as collagen I, osteopontin, osteocalcin, and osteonectin. Additionally, it contained growth factors like FGF-2, HGF, and lower levels of VEGF. The study demonstrated strong adhesion and sustained viability of prostate cancer cells, with PC3 and LNCaP cells exhibiting morphology and organization reminiscent of bone tissue [120]. These findings demonstrate the fruitful incorporation of cell-derived matrices and engineered scaffolds for successful 3D bone tumor modeling.

### 5.3 Tissue engineering constructs and scaffolds

Scaffolds are essential in 3D bone tumor modeling, providing structural support that mimics the native ECM for cancer cell interactions. They can be derived from natural or synthetic materials (Table 1, Table 2), each with distinct advantages. Natural scaffolds offer superior biocompatibility but may suffer from mechanical weakness and variability, while synthetic scaffolds provide consistency and customizable properties but may lack biological functionality. An ideal scaffold must balance mechanical strength, biocompatibility, and functional cues to support cancer cell adhesion, proliferation, and migration [12,121].

**Table 1:** Natural and synthetic polymers incorporated in 3D bone tumor models.

**Type**	**Biomaterial**	**3D structure**	**Cancer**	**Refs**
Natural	Collagen	Scaffold, hydrogel	Ewing’s sarcoma, osteosarcoma	[65,88,136]
	Hyaluronic acid	Scaffold, hydrogel	Ewing’s sarcoma, prostate cancer	[65,150]
	Gelatin	Hydrogel	Breast cancer	[90]
	Chitosan	Scaffold, hydrogel	Breast cancer, prostate cancer	[94,136]
	Silk fibroin	Scaffold	Breast cancer	[142,143]
	Resin	Scaffold	Breast cancer	[159]
Synthetic	PPF	Scaffold	Ewing’s sarcoma	[72]
	PCL	Scaffold	Ewing’s sarcoma, prostate cancer	[73,157,158]
	PEG	Scaffold, hydrogel	Breast cancer, osteosarcoma	[84,85,160]
	PU	Scaffold	Osteosarcoma	[86]
	PLGA	Scaffold	Breast cancer	[93]
	GelMA	Scaffold, hydrogel	Osteosarcoma, breast cancer	[112,161]

**Abbreviations**.

PFF; poly-propylene fumarate, PCL; poly(ε-caprolactone), PEG; polyethylene glycol, PU; poly urethane, PLGA; poly(lactide-co-glycolide), GelMA; gelatin methacryloyl.

**Table 2:** Overview of 3D *in vitro* bone cancer models, their relevance, and core attention.

**3D model**	**Cancer**	**Relevance**	**Core attention**	**Refs**
Collagen 1-hyaluronic acid hydrogel	Ewing’s sarcoma	Mechanical stimulation enhanced cancer progression and reduced the effectiveness of sorafenib.	C	[65]
GAG or nanohydroxyapatite-incorporated collagen scaffolds	Prostate cancer	PC3 cells exhibited lower proliferation rates on nanohydroxyapatite-containing scaffolds, while LNCaP cells demonstrated increased PSA secretion, particularly in GAG-containing scaffolds.	Trop	[136]
PPF scaffold	Ewing’s sarcoma	Scaffolds with smaller pores experienced higher shear stress due to increased fluid resistance, while flow perfusion improved cell viability and IGF-1 production.	TME	[72]
PEGDA hydrogel	Osteosarcoma	Considerable integration and colonization in ECM-enriched scaffolds were demonstrated. Cancer cells need a high stiffness (50 kPa) environment for growth.	Trop, BIO	[85,86]
PEG/PEGDA hydrogel	Breast cancer	Robust attachment and spreading, with the highest proliferation rate, were observed on small square-patterned matrices.	Trop	[84]
PCL scaffold	Ewing’s sarcoma	Higher flow rates significantly enhanced cell growth and proliferation as well as resistance to dalotuzumab. The porous scaffolds enhance cancer drug resistance.	C	[73,158]
hMSCs-seeded calcium phosphate- miniaturized PCL scaffold	Prostate cancer	hMSCs seeded on the scaffold supported cancer tumoroid growth as well as chemoresistance.	C	[157]
Microfluidics co-cultured endothelial cells with MSCs	Breast cancer	Densely interconnected vessel-like structures were developed. The presence of flow resulted in a fourfold reduction in cancer cell growth. The CXCL5/CXCR2 signaling significantly enhanced breast cancer cell extravasation.	Met, Trop	[68,167]
Microfluidics cultured of spheroids combining PC-3 cells, osteoblasts, and endothelial cells	Prostate cancer	Cancer cells remained quiescent within over seven days and proliferated significantly slower in the 3D spheroids compared to 2D cultures, more closely mirroring *in vivo* cancer growth patterns.	Met	[168]
Gelatin-transglutaminase hydrogel	Breast cancer	Higher circumferential and radial stresses in stiffer environments slow cell proliferation.	BIO	[90]
Hydroxyapatite-incorporated PLGA scaffold	Breast cancer	Smaller, less crystalline hydroxyapatite enhanced cancer cell adhesion and proliferation.	Trop	[93]
Cell-derived matrix	Breast cancer	The matrix composed primarily of ECM proteins supported osteoblast maturation, significantly impacting metastatic breast cancer cell colonization.	Met	[118]
Hydroxyapatite-incorporated chitosan hydrogel	Breast cancer	Hydroxyapatite size, crystallinity, and concentration significantly influence breast cancer cell adhesion.	Trop	[94]
Human primary osteoblast-derived matrix	Breast cancer	Supported breast cancer cell adhesion and proliferation but also influenced key metastatic behaviors.	Trop	[119]
Integrin-binding and MMP-degradable peptides-functionalized hyaluronan-based hydrogel	Prostate cancer	The hydrogel supported both the survival and expansion of cancer cells co-cultured with osteoblastic cells.	TME	[150]
hMSCs-seeded decellularized bone scaffolds	Ewing’s sarcoma	Supported key tumor characteristics, including hypoxic and glycolytic phenotypes.	BIO	[166]
MSCs-seeded Fibronectin-coated 3D-printed resin	Breast cancer	Facilitated colonization, survival, and proliferation of cancer cells and limited their sensitivity to cisplatin.	C	[159]
Nanohydroxyapatite and MSCs-incorporated 3D bioprinted GelMA	Breast cancer	Cancer cells showed a preference for higher GelMA concentration to proliferate.	Trop	[161]
Silk fibroin scaffolds	Breast cancer	Cancer cells co-cultured with osteoblast-like cells exhibit increased resistance to paclitaxel.	C	[143]
Star-PEG-heparin hydrogel	Breast cancer	The hydrogel enabled the binding of adhesion-mediating peptides as well as controlled liberation of signaling molecules.	TME	[160]

**Abbreviations**.

PFF; poly-propylene fumarate, IGF-1; insulin-like growth factor-1, MSCs; mesenchymal stem cells, PEG; polyethylene glycol, PEGDA; PEG-diacrylate, CXCL5; C‐X‐C motif chemokine ligand 5, CXCR2; CXC receptor 2, PLGA; poly(lactide-co-glycolide), GAG; glycosaminoglycans, PSA; prostate-specific antigen, MMP; matrix metalloproteinase, PCL; poly(ε-caprolactone), GelMA; gelatin methacryloyl, C; chemo-response, TME; tumor microenvironment, Met; dormancy/metastatic behavior, Trop; bone tropism, BIO; Cancer biology.

Soft tissue matrix models, particularly hydrogels, are increasingly used to replicate the 3D ECM of solid tumors such as breast, prostate, and lung cancers [[122], [123], [124]]. As hydrophilic polymer networks, hydrogels can mimic the viscoelastic properties of soft tissues. Natural hydrogels, composed of ECM proteins like collagen, laminin, and fibrin, support cell attachment, proliferation, and differentiation due to their biodegradability and cell-binding motifs [125]. Synthetic hydrogels, such as PEG and PVA, offer tunable mechanical properties, structural consistency, and bioinertness, making them ideal for long-term applications [126,127]. By incorporating bioactive elements like integrin-binding peptides and growth factors, synthetic hydrogels can effectively guide cell behavior, closely simulating native ECM functions [127]. Hydrogels often fail to replicate the mechanical properties of bone, limiting their ability to study bone tumor behavior accurately [128]. Hybrid approaches, combining natural hydrogels with synthetic materials, enhance structural support by integrating the mechanical strength of synthetics with the cell-friendly environment of natural components, optimizing conditions for 3D cell growth and adhesion [129,130].

#### 5.3.1 Matrigel

Matrigel, a basement membrane extract derived from Engelbreth-Holm-Swarm mouse sarcoma, is rich in ECM proteins such as laminin, collagen IV, and heparan sulfate proteoglycan, along with growth factors like EGF, TGF-β, and PDGF. Its composition closely mimics the natural basement membrane, making it a widely used substrate for 3D cell culture and tumor biology studies [131]. Laminin and collagen IV facilitate essential processes like cell adhesion and migration, which are critical for angiogenesis and cancer progression [132]. However, Matrigel's batch-to-batch variability necessitates careful selection and characterization to ensure experimental reproducibility and relevance to *in vivo* conditions [133]. Mano et al. developed hydrogel-based 3D *in vitro* models using GelMA 10 % and Matrigel to examine their effects on osteosarcoma cell invasion. Their study demonstrated that cells in the softer Matrigel began migrating on the first day, while those in the mechanically robust GelMA 10 % initiated migration on the third day. These findings highlight the critical influence of matrix stiffness on cancer cell invasion, supporting previous research by Lam et al. on the impact of hydrogel stiffness on tumor cell behavior in 3D spheroids [112,134].

#### 5.3.2 Collagen

Collagen, the primary protein constituent of bone, comprises approximately 10 % of the bone matrix and serves as a versatile material for bone applications [135]. Collagen-based hydrogels, inherently low in stiffness, can be engineered and cross-linked to enhance their mechanical properties [136]. Incorporating nanohydroxyapatite, which mimics bone’s inorganic mineral phase, significantly improves osteoinductivity and mechanical strength [137]. Additionally, glycosaminoglycans (GAGs) are integrated to enhance cell attachment, proliferation, and differentiation, making collagen-based scaffolds highly effective for bone-related applications [138]. Fitzgerald et al. evaluated the growth of PC3 and LNCaP prostate cancer cells on three collagen-based scaffolds designed to replicate the bone microenvironment. These scaffolds varied in composition, incorporating either collagen alone or collagen combined with GAG or nanohydroxyapatite. PC3 cells exhibited lower proliferation rates on nanohydroxyapatite-containing scaffolds, aligning with nanohydroxyapatite’s role in mimicking bone hardness, which may influence cell proliferation dynamics. Conversely, LNCaP cells demonstrated increased prostate-specific antigen (PSA) secretion, particularly in GAG-containing scaffolds, underscoring GAG’s role in enhancing cellular function and matrix interaction [136].

#### 5.3.3 Silk fibroin

Silk fibroin scaffolds, derived from fibroin fibers in raw silk, can be engineered into various forms, such as hydrogels and films, with tunable properties, including pore size, cross-linking, mechanical strength, and surface roughness [139,140]. These characteristics make silk highly valuable for biomedical applications, particularly in bone metastasis research. Its adaptability allows for the integration of features like cellular recognition and mineralization, making it a promising material for 3D bone modeling, where strength, biocompatibility, and porosity are essential [141]. Silk fibroin scaffolds have been effectively utilized as 3D matrices to support the growth and interaction of human breast adenocarcinoma and osteoblast-like cells, demonstrating their potential in modeling bone-breast cancer metastasis. Specifically, fibroin derived from the non-mulberry silkworm *Antheraea mylitta* provided a cytocompatible and structurally favorable environment for cell attachment and proliferation [142]. Additionally, studies on 3D silk-based metastasis models confirmed that silk fibroin scaffolds effectively facilitate interactions between breast cancer cells and osteoblast-like cells, enabling the study of cancer cell invasiveness and angiogenesis. Notably, the spatial arrangement of cells within the silk scaffolds plays an important role in modulating these interactions, further emphasizing their relevance in bone metastasis research [143].

#### 5.3.4 Chitosan

Chitosan, derived from chitin, is widely recognized for its biodegradability and non-toxicity, making it a valuable material in tissue engineering applications, particularly for bone regeneration [144,145]. While inherently osteoconductive, chitosan requires modifications to enhance its mechanical properties and biological activity for effective use in hard tissue engineering [146]. To address these limitations, chitosan is often reinforced with materials such as silk, alginate, gelatin, or ceramics like tricalcium phosphate and hydroxyapatite [147]. Zhu et al. investigated a biomimetic bone model utilizing hydroxyapatite/chitosan scaffolds optimized with nano-sized hydroxyapatite and MSC-modified bioactive materials. This approach promoted deeper cell penetration and higher cell density, particularly for the highly metastatic MDA-MB-231 breast cancer cells compared to the less metastatic MCF-7 cells. The MSC modification involved osteogenic differentiation, during which MSCs deposited bioactive components, including proteins and extracellular calcium, followed by a decellularization process. Furthermore, co-culturing breast cancer cells with MSCs led to significant upregulation of the MTDH gene, a key regulator of cancer proliferation and chemoresistance. These findings underscore the scaffold's potential not only in replicating bone microenvironment interactions but also in influencing cancer cell behavior [94].

#### 5.3.5 Hyaluronic acid

**Fig. 6:**
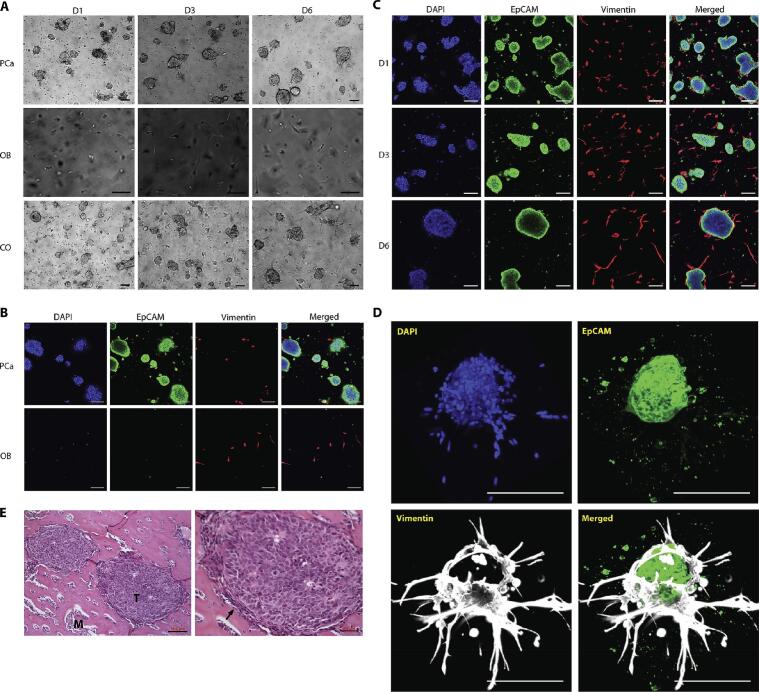
Organization of PCa and osteoblastic cells in co-culture. (A) MDA PCa 118b monocultures (PCa), MC 3 T3-E1 monocultures (OB), and their co-cultures (CO) at different time points. (B and C) EpCAM-positive (green) tumor cells and vimentin-positive (red) osteoblastic cells in monocultures (B) and co-cultures (C) at day 6. (D) A 3D volume rendering of an osteoblast-wrapped PCa tumoroid in co-culture. (E) Hematoxylin-eosin-stained sections of an intrafemorally grown MDA PCa 118b PDX. T (tumor), M (bone matrix); the black arrow marks osteoblasts. Adapted with permission [150]. Copyright 2016, Elsevier. (For interpretation of the references to colour in this figure legend, the reader is referred to the web version of this article.)

Hyaluronic acid, a key structural component of the ECM, is widely recognized for its exceptional biocompatibility and non-toxicity [148]. Hyaluronic acid's capacity for cross-linking enables the fabrication of hydrogels with tunable mechanical properties, allowing for precise customization to meet specific tissue engineering requirements [149]. Fong et al. developed a bone tumor microenvironment model by co-encapsulating patient-derived xenograft prostate cancer tumoroids and MC3T3-E1 osteoblastic cells within a 3D hyaluronan-based hydrogel. This hydrogel was functionalized with integrin-binding and MMP-degradable peptide sequences to enhance cell-matrix interactions and more accurately recapitulate the bone metastatic niche. Within this engineered microenvironment, osteoblastic cells exhibited spreading behavior, while prostate cancer tumoroids formed compact aggregates, with osteoblasts extending toward the tumoroids—closely resembling *in vivo* tumor-surrounding architecture. The study sought to assess whether the 3D hydrogel could sustain prostate cancer cell viability and proliferation, a challenge in conventional 2D cultures. Results demonstrated that the hydrogel effectively supported both the survival and expansion of prostate cancer cells co-cultured with osteoblastic cells (Fig. 6) [150].

#### 5.3.6 PEG

PEG is a highly hydrophilic, inert synthetic polymer with tunable mechanical properties and processability, including advanced fabrication techniques such as 3D printing. By adjusting the degree of cross-linking, PEG-based materials can be engineered to achieve specific stiffness and porosity levels, making them highly adaptable for scaffold fabrication in tissue engineering [151]. A common modification involves acrylation to form PEGDA, facilitating hydrogel formation with controlled mechanical characteristics. Despite its versatility, PEG lacks intrinsic bioactive domains, necessitating modifications to enhance biological interactions. To address this limitation, studies have explored strategies to optimize PEG-based matrices for complex tissue engineering applications [152]. Jabbari et al. demonstrated that PEGDA hydrogels could be precisely tuned to stiffness levels ranging from 2 to 70 kPa, facilitating controlled studies on CSC behavior, including growth and marker expression [85]. Additionally, a bioprinted PEG scaffold revealed that the stiffness of the substrate could influence the migration speed of malignant cells [153]. Further, Zhu et al. incorporated hydroxyapatite nanoparticles into PEG/PEGDA matrices to create a biomimetic microenvironment conducive to cancer cell proliferation, highlighting the potential of PEG-based systems in tumor modeling and regenerative medicine [84].

#### 5.3.7 PCL

PCL is an FDA-approved, biocompatible polymer, particularly used for 3D bone tumor modeling due to its favorable mechanical properties, structural stability, and non-toxic nature [154]. Its tunable stiffness and ability to integrate with other biomaterials make it highly suitable for scaffold fabrication with precise fiber diameters. Additionally, surface modifications, such as incorporating RGD sequences, enhance its bioactivity, further supporting its application in complex scaffold structures [155]. PCL-based scaffolds have been widely used to replicate bone microenvironments and advance cancer research. Shokoohmand et al. developed a 3D osteoblast-derived construct using medical-grade PCL (mPCL) scaffolds coated with calcium phosphate, which promoted osteogenic differentiation and the formation of a mineralized ECM resembling human bone [156]. Paindelli et al. also seeded hMSCs on a miniaturized medical grade, calcium phosphate PCL scaffold and demonstrated its role in supporting prostate cancer tumoroid growth as well as chemoresistance, similar to *in vivo* evidence demonstrating that osteoblasts protect cancer cells against chemo-therapeutics [157]. Fong et al. developed electrospun 3D PCL scaffolds as *ex vivo* 3D Ewing’s sarcoma models, mimicking the morphology, growth kinetics, and protein expression of human tumors. Results of culturing cancer cells in the porous 3D scaffold showed enhanced drug resistance and altered IGF-1R/mTOR signaling compared to 2D cultures [158]. These findings highlight the potential of PCL scaffolds in developing physiologically relevant cancer models and improving therapeutic studies.

### 5.4 Native bone scaffolds

Decellularized bone matrixes have emerged as valuable scaffolds for bone cancer modeling, offering a closer replication of the native ECM composition and structure. Decellularized ECM retains the biochemical and structural modifications induced by tumor growth, reinforcing its relevance in studying tumor-matrix interactions [162]. Although it lacks precise control over matrix parameters, the decellularization process preserves key ECM proteins while removing cells, enabling the controlled reseeding of specific cell types for further investigation [163].

Rusyn et al. developed a reproducible bone cancer model using a decellularized bone matrix seeded with osteogenically differentiated hMSCs and infused with Ewing’s sarcoma cell spheroids. The model revealed distinct drug interactions, with doxorubicin binding significantly to the scaffold while linsitinib exhibited rapid loss, mirroring its clinical elimination. Additionally, doxorubicin altered the bone microenvironment by increasing osteopontin (OPN) levels, while dexamethasone, typically non-toxic in 2D cultures, reduced tumor viability in 3D models. Transcriptome analysis demonstrated high reliability, with 84 % consistency across experiments [164,165]. Villasante et al. used decellularized bone scaffolds seeded with hMSCs to create engineered bone tissue, validated by high expression of bone markers such as bone sialoprotein (BSP), OPN, and osteocalcin (OCN). Integrating Ewing’s sarcoma spheroids into these constructs enabled the study of tumor-bone interactions, closely mirroring gene expression profiles of actual tumors. The models exhibited key tumor characteristics, including hypoxic and glycolytic phenotypes, supporting their potential for cancer research and drug evaluation [166].

## 6 Crosstalk between cancer cells and bone microenvironment

### 6.1 Tumor-stroma interactions

The bone microenvironment contributes to cancer progression by influencing the genetic and phenotypic evolution of cancer cells through interactions with stromal cells and the ECM. Effective therapeutic strategies must account for the dynamic relationship between cancer cells and the stroma; however, replicating the metastatic process in 3D models remains challenging due to the complexity of bone metastatic niches. Bone stromal cells, including osteoblasts, osteoclasts, and MSCs, interact with cancer cells via direct contact, gap junctions, cytokines, and extracellular vesicles [[169], [170], [171], [172]]. Numerous studies have highlighted the impact of bone microenvironment on cancer progression, metastasis, and drug resistance. Breast and prostate cancer cells interact dynamically with bone stromal cells, modifying their behavior and metastatic potential. Conditioned media experiments revealed reduced breast cancer cell viability and attachment when exposed to osteoblast-like cell-conditioned media, while osteoblasts experienced reduced viability and morphological changes upon exposure to breast cancer-conditioned media [143]. Similarly, prostate cancer cells induce significant genetic and phenotypic changes in bone stromal cells, enhancing their capacity to support tumor growth *in vivo* [173]. Further, in certain conditions, tumor cells might acquire bone-like characteristics, a phenomenon called osteomimicry, facilitating their survival in the bone niche. This is observed in prostate and breast cancers, where tumor cells express bone matrix proteins and osteoblast-related genes [174,175]. Advanced biomimetic 3D *in vitro* models help replicate tumor-stroma interactions, introducing critical factors that influence tumor growth and invasiveness, like TGF-β1 and SDF-1 for breast cancer and FGFR1 for prostate cancer [150,160].

Drug resistance is a major challenge within the bone-tumor microenvironment. Tumor-stroma co-cultures enhance resistance to chemotherapy, such as paclitaxel in breast cancer and IGF-1R inhibitors in Ewing’s sarcoma. Findings based on 3D *in vitro* models have revealed that this resistance might be linked to signaling pathways like IL-6/STAT3, as well as efflux transporters upregulation and apoptosis-related proteins alteration. Such information helps find important therapeutic targets to address undesired tumor-stroma interactions [143,[176], [177], [178], [179]]. Further, the spatial and temporal evolution of bone metastases is crucial in understanding their complexity. Sequentially engineered 3D scaffolds that mimic the progressive development of the bone-tumor microenvironment can simulate escalating chemoresistance and altered cellular behaviors [177].

### 6.2 Tumor dormancy and metastasis

Cancer cells infiltrate bone through the trabecular bone's slow-flowing vascular sinusoids. Once inside, they may either proliferate into metastatic lesions or enter dormancy in a cell-cycle arrest state. Their fate depends on the local microenvironment, which can either promote growth or maintain dormancy, and only a small fraction successfully forms stable metastases [180,181]. Dormant cells may later activate under stress, immune suppression, or molecular stimulation, potentially leading to relapse even decades after treatment [182]. Eliminating dormant tumor cells is essential for long-term remission and overcoming therapy resistance.

Researchers have developed advanced 3D *in vitro* models to study cancer cell interactions with the bone microenvironment, particularly the mechanisms of metastasis and dormancy. Hao et al. designed a bone-on-a-chip microfluidic device that mimics bone metastasis. In this system, MDA-MB-231 metastatic breast cancer cells invaded and disrupted osteoblastic tissue, whereas metastasis-suppressed MDA-MB-231-BRMS cells remained dormant [183]. Marlow et al. created a 3D *in vitro* model simulating inhibitory and supportive bone niches. Breast cancer cells in the inhibitory niches entered reversible cell-cycle arrest without dying, and upon transfer to a supportive niche, they resumed proliferation. This dormancy was regulated by signaling pathways such as p38 MAPK, Alk5, and RTKs [180]. Mastro et al. used a bioreactor-based model with mineralized osteoblast tissue to investigate dormancy. They found that dormant cancer cells could be reactivated by bone remodeling cytokines (TNFα and IL-1β), a process mediated by prostaglandins. Inhibiting prostaglandin pathways suppressed this transition, emphasizing their role in dormancy escape [184].

### 6.3 Osteolytic and osteoblastic lesions

The bone microenvironment plays a crucial role in maintaining homeostasis but becomes a key factor in pathological conditions such as bone metastasis, where cancer cells disrupt normal regulatory mechanisms to establish metastatic sites. Metastatic bone disease manifests along a spectrum from osteolytic to osteoblastic lesions [185]. Breast cancer metastases are predominantly osteolytic, driven by tumor-secreted parathyroid hormone-related protein (PTHrP), IL-8, and IL-11, which enhance RANK-L production, stimulating osteoclastogenesis and bone resorption. This process releases growth factors like TGF-β and IGFs, fueling a feedback loop that perpetuates osteolysis [[186], [187], [188]]. Additionally, *in vitro* studies using 3D co-culture models reveal that breast cancer cells cluster around osteoblast-like cells, impairing their function and mineralization [143]. However, 15–20 % of breast cancer patients develop osteoblastic lesions as a compensatory response to bone loss [189]. In contrast, prostate cancer metastases are largely osteoblastic, characterized by abnormal bone deposition driven by tumor-derived BMPs, TGF-β, and IGF-1. Despite increased bone formation, these lesions are structurally weak and prone to fractures. Notably, even osteoblastic lesions exhibit increased bone resorption [[189], [190], [191]]. Osteosarcoma cells also contribute to bone degradation by releasing factors that promote osteoclast differentiation, leading to enhanced bone resorption and tumor progression [[192], [193], [194], [195]].

### 6.4 Bone tropism and pre-metastatic niche

Bone metastasis is supported by the bone tissue's microenvironment rich in chemokines and growth factors, thereby promoting tumor growth and invasion [196,197]. Moretti et al. engineered a bone microenvironment containing osteoblasts, osteoclasts, and endothelial cells within a fibrin matrix and compared it with a muscle-like environment. Cancer cells proliferated more in bone than in muscle, emphasizing bone's preferential role in metastasis [198]. Cancer cells adhere to the bone matrix through integrins like αvβ3 and αvβ5, which bind to bone-specific ECM proteins, facilitating colonization. While αvβ3 integrin does not drive tumor proliferation, it enhances adhesion, migration, and osteoclast recruitment, increasing bone resorption [199,200]. Accordingly, targeting αvβ3 integrin is a potential strategy to limit breast cancer bone metastasis. Taubenberger et al. demonstrated that metastatic breast cancer cells strongly adhere to human primary osteoblast-derived matrices via β1-integrins [119]. Similarly, PC3 prostate cancer cells expressing collagen I integrin receptors exhibited enhanced adhesion and proliferation on collagen-based 3D scaffolds [136].

Furthermore, multiple studies have highlighted the role of osteoblast-derived signals in bone metastasis. Talukdar et al. observed that breast cancer cells undergo chemotactic migration toward osteoblast-like constructs in 3D silk fibroin scaffolds [143]. In alignment with these findings, Bray et al. developed a biphasic 3D scaffold model integrating hydrogels and cryogels to simulate bone metastasis, where breast cancer cells migrated into osteoblast-containing cryogels, forming distinct morphological structures [160]. The interaction between osteoblasts, bone marrow stromal cells, and cancer cells is largely driven by chemokine signaling, particularly the CXCL12/CXCR4 and CX3CL1/CX3CR1 axes. Osteoblasts and stromal cells in the bone marrow secrete CXCL12, creating a chemotactic gradient that attracts CXCR4-expressing tumor cells, facilitating prostate and breast cancer cell homing to the bone [[201], [202], [203]]. Similarly, CX3CL1 produced by osteoblasts and endothelial cells aids in the retention of cancer cells via CX3CR1 receptors [204,205]. To model these interactions, several advanced *in vitro* platforms have been developed. Keller et al. introduced a macrofluidic model that connects primary and metastatic tumor sites using real bone tissues. Prostate cancer cells migrated from a primary tumor site to a bone chip, guided by CXCL12. Blocking CXCR4 with AMD3100 reduced bone-directed migration, confirming the CXCL12/CXCR4 axis's role in metastasis [206]. Bersini et al. developed a 3D microfluidic model incorporating osteo-differentiated MSCs and endothelial cells to simulate a vascularized bone niche. The study demonstrated that CXCL5/CXCR2 signaling significantly enhances breast cancer cell extravasation, a key step in metastasis [167]. Collectively, these studies emphasize the significance of 3D models in recapitulating the bone microenvironment, elucidating key molecular interactions driving metastasis, and identifying potential therapeutic targets.

Beyond being a favorable site for metastasis, the bone microenvironment can be preconditioned by primary tumors through pre-metastatic niche formation. Tumors remotely alter the bone environment by releasing cytokines, growth factors, and extracellular vesicles (EVs), particularly exosomes, which carry oncogenic molecules that modify bone marrow MSCs to support metastasis [[207], [208], [209], [210]]. Notably, prostate cancer-derived exosomes containing pyruvate kinase M2 (PKM2) upregulate CXCL12 in bone marrow MSCs, which enhances tumor cell seeding and proliferation in the bone marrow [211]. microRNAs (miRNAs) also play a vital role in bone metastasis by promoting osteoclastogenesis and bone resorption, creating a favorable environment for tumor colonization. Exosomal miR-21 levels are significantly higher in breast cancer patients with bone metastases, suggesting its potential as a biomarker for diagnosis and risk assessment. Additionally, exosomes from highly metastatic breast cancer cells enhance osteoclast activity and bone degradation, facilitating pre-metastatic niche formation [212]. MiR-218, another key miRNA, disrupts bone homeostasis by reducing type I collagen production in osteoblasts, further promoting a bone microenvironment conducive to metastasis [213].

## 7 Drug delivery using 3D *in vitro* models

3D *in vitro* models provide a more physiologically relevant alternative to traditional 2D cell cultures, better replicating the TME by incorporating spatial and mechanical characteristics like *in vivo* tumors, making them essential for studying drug efficacy and delivery. By allowing researchers to assess how therapies, especially nanoparticle-based systems, penetrate and interact with tumor-like structures, 3D models bridge the gap between conventional cell cultures and *in vivo* studies, advancing drug development and precision medicine. Charoen et al. developed uniform tumor spheroids with live cells at the periphery and necrotic cores, mirroring real tumors, and showed that paclitaxel-loaded expansile nanoparticles significantly reduced spheroid size more effectively than conventional treatment [88]. Fitzgerald et al. investigated siRNA delivery in prostate cancer–osteoblast co-culture using a collagen-based 3D scaffold. A cationic cyclodextrin-based vector was used for siRNA transfection. Notably, gene knockdown was less efficient in 3D cultures compared to 2D cultures, suggesting upregulated resistance mechanisms in a bone-mimetic environment [214]. Subia et al. utilized silk fibroin 3D scaffolds to co-culture breast cancer and osteoblast-like cells, assessing doxorubicin-loaded nanoparticles for targeted drug delivery. Folate-conjugated silk fibroin nanoparticles selectively reduced breast cancer cell proliferation without harming osteoblasts, preserving bone integrity. Additionally, the treatment decreased angiogenesis and metabolic activity, demonstrating the efficacy of targeted drug delivery in metastatic bone environments [142]. Together, these studies emphasize the value of 3D models in replicating real tumor–bone interactions and advancing precision drug delivery strategies.

## 8 Emerging technologies

### 8.1 Integration of machine learning and computational modeling

The integration of machine learning into cancer research is transforming our ability to model disease dynamics and predict pathological outcomes [215,216]. For instance, Xiong et al. employed temporal variational autoencoders (T-VAEs) to analyze longitudinal micro-CT images in murine models of breast cancer bone metastasis. This approach successfully forecasted osteolytic lesion progression by capturing temporal degradation patterns, offering a robust tool for early diagnosis and therapeutic monitoring [217]. In parallel, cancer-on-a-chip platforms are redefining 3D *in vitro* modeling by enabling high-throughput, physiologically relevant drug testing [218,219]. These microfluidic systems allow precise simulation of the bone-tumor interface and support multiplexed analyses across thousands of miniaturized wells, as demonstrated by Zhang et al. [218,220]. The resulting high-dimensional datasets necessitate advanced computational infrastructure. Here, machine learning plays a pivotal role, facilitating real-time data acquisition, efficient storage, and predictive modeling of drug responses and tumor behavior [221]. Together, machine learning and cancer-on-a-chip technologies form a synergistic framework for accelerating bone cancer research and preclinical drug evaluation.

Computational modeling has become a critical tool in bone cancer research, helping better understand tumor progression, treatment response, and bone microenvironment dynamics [90,222,223]. These models facilitate hypothesis testing, optimize therapeutic strategies, and reduce the dependency on costly *in vivo* trials. In prostate cancer bone metastasis, simulations have identified the pre-metastatic phase as the optimal window for TGFβ inhibition, and the findings were validated in both animal models and patient-derived bone samples. Additionally, patient-specific modeling has enabled the prediction of individualized treatment outcomes [224]. Another investigation simulated bone matrix homeostasis and its disruption by metastatic lesions, revealing that while bisphosphonates slow cancer progression, anti-RANKL therapy may fully eliminate metastases under defined conditions [223]. Recently, Kumar et al. developed a 3D *in vitro* model that integrates computational simulations to investigate the interplay between mechanical loading and tumor-induced bone degradation. Using a custom bioreactor, they demonstrated that mechanical stimulation could attenuate the osteolytic activity of breast cancer cells. Computational analysis enabled the prediction of cell responses to biomechanical cues, offering mechanistic insights into how physical forces within the bone microenvironment influence tumor progression and bone resorption [90]. These findings underscore the potential of combining computational frameworks with biomimetic 3D platforms to personalize and refine bone-targeted therapies in metastatic cancer.

### 8.2 Integration of multi-omics and systematic biology

The emergence of 3D *in vitro* models represents a major advancement over traditional 2D cultures, providing more accurate chemosensitivity profiles crucial for drug discovery and personalized medicine by closely reflecting proteomic and genetic signatures found in actual tumors. However, their full potential can only be realized through integration with genomic, proteomic, and metabolomic profiling, allowing researchers to decode the intricate molecular networks driving tumor progression. A study by Hanjun Li et al. compared gene expression in osteosarcoma cells across 2D cultures, tumor spheroids (CSCs), and a 3D bioprinted model using GelMA/HAMA hydrogel. The 3D model, designed with a grid-like scaffold, facilitated nutrient exchange and waste removal, closely mimicking the bone tumor microenvironment. Cells in this system exhibited a distinct gene expression profile, with altered DNA replication, amino acid degradation, and cell adhesion pathways, alongside reduced proliferation and modified cellular interactions. Multi-omics analysis revealed significant epigenetic modifications, particularly in autophagy, amino acid degradation, and cellular adhesion pathways. Notably, osteosarcoma cells in the 3D model showed enhanced autophagy, as evidenced by increased expression of autophagy-related genes, a phenomenon linked to tumor progression and poor prognosis. Using an osteosarcoma tissue microarray, the study found higher LC3 expression in late-stage tumors, suggesting a critical role for autophagy in disease progression. Moreover, the 3D model’s heightened sensitivity to autophagy inhibitors emphasized the importance of 3D culture systems in refining cancer treatment strategies [225].

### 8.3 Incorporation of immune cells or adipocytes in 3D models

The incorporation of immune cells and adipocytes into 3D *in vitro* tumor models significantly enhances their ability to replicate the bone tumor microenvironment. These models provide a more comprehensive understanding of tumor progression, particularly in the context of bone metastases, while also offering valuable platforms for evaluating the efficacy of immunotherapies and metabolic-targeted treatments. Immune cells such as macrophages, T-cells, and natural killer (NK) cells play dual roles in tumor development, either promoting or inhibiting metastasis [226]. Their inclusion in 3D culture systems allows for detailed investigation of immune-tumor interactions and the effectiveness of immunotherapeutic strategies. A study by Moretti et al. engineered a bone microenvironment model to study breast cancer bone metastases, creating metastatic immune bone minitissue by combining bone minitissue, including osteoblasts, osteoclasts, and endothelial cells within a fibrin matrix, with cancer cells and macrophages. Increased IL-20 levels, as well as active bone remodeling markers (MMP2 and OPN), were observed. Cancer cells influenced macrophage polarization to an M2-like tumor-associated macrophage phenotype, driven by increased IL-10 secretion, reduced inflammatory regulators (sTNFR1, sTNFR2), and enhanced A proliferation-inducing ligand (APRIL) production, creating a pro-metastatic microenvironment [198].

Adipocytes are abundant in bone marrow, a common site for cancer metastasis. They secrete cytokines, growth factors, and fatty acids that promote tumor survival, invasiveness, and therapy resistance. Recent studies highlight that bone marrow adipocytes support tumor growth through fatty acid transfer and adipokine signaling, making them critical players in the bone metastatic niche [227,228]. A study by Herroon et al. explored the interaction between prostate cancer cells and bone marrow-derived adipocytes in two 3D culture systems. In spheroid-based culture, prostate tumor cells formed larger, more disorganized spheroids in the presence of adipocytes, exhibiting increased proliferation and enhanced glycolytic metabolism. In collagen I matrix culture, tumor cells migrated towards and integrated with adipocytes, promoting tumor invasion and ECM degradation. Macrophage interaction studies revealed that tumor spheroids co-cultured with adipocytes attracted more bone marrow macrophages, further altering tumor growth dynamics [229]. These advanced models provide crucial insights into tumor-immune-metabolic interactions, paving the way for more effective and targeted therapies for bone-metastatic cancers.

In comparison, humanized mouse models, immunodeficient mice co-engrafted with human tumors and immune components, offer a physiologically relevant *in vivo* platform for studying complex tumor-immune interactions. These models enable a comprehensive assessment of systemic immune responses, pharmacokinetics, and potential immune-related adverse effects associated with immunotherapies [230]. They have been instrumental in evaluating the efficacy of immune checkpoint inhibitors, e.g., anti-PD-1 and anti-CTLA-4 therapies, in a setting that closely mimics human physiology [231,232]. However, challenges persist, including incomplete immune system reconstitution and potential discrepancies in immune cell functionality, possibly given the human donor variability [230]. Notably, engineered 3D *in vitro* models have also been successful in evaluating the therapeutic potential of immune checkpoint inhibitors in bone cancers [233,234].

### 8.4 Integrating Patient-Derived xenografts and primary cell lines

Patient-derived xenograft (PDX) models and primary cell lines both serve as valuable tools for 3D *in vitro* cancer modeling, each with distinct advantages and limitations. PDX models, established by implanting human tumor tissues into immunodeficient mice, closely preserve the tumor's genetic, cellular, and phenotypic characteristics, making them highly accurate for studying tumor biology, progression, metastasis, and drug response [235,236]. They enable “mouse clinical trials” to refine treatments before human trials and facilitate personalized medicine through genomic integration [237]. However, their translational relevance is limited due to the replacement of human stromal cells with murine ones, their inability to model immune responses, and the high costs, time requirements, and ethical considerations associated with their use [[238], [239], [240]]. Conversely, primary cell lines, derived directly from patient tumors and cultured *in vitro*, retain many of the tumor’s genetic and functional properties. In 3D cultures, they better replicate TME than traditional 2D models, making them effective for drug testing and mechanistic studies [237,241]. However, their limited lifespan, need for frequent derivation, and variability across donors present challenges in standardization and reproducibility [242]. Additionally, maintaining their original characteristics requires stringent cultural conditions, technical expertise, and adherence to ethical regulations regarding patient consent and the use of biological materials [[243], [244], [245]].

Paindelli et al. investigated bone metastasis-derived PDX models (MDA PCa 183 and MDA PCa 118b) in a bone-mimetic environment using polycaprolactone scaffolds coated with calcium phosphate. The aggressive, androgen-independent MDA PCa 118b cells thrived exclusively in the bone-mimetic environment, maintaining their compact epithelial structure. In contrast, the androgen-dependent MDA PCa 183 cells required stromal cell support within the bone-mimetic environment to form colonies, highlighting the role of the bone-like environment in sustaining metastatic prostate cancer cells *in vitro* [157]. Fong et al. developed a 3D bone TME model by embedding MDA PCa 118b tumoroids and osteoblastic cells in hyaluronic acid hydrogel with integrin-binding and MMP-degradable sequences. The co-cultured cells retained tumor characteristics and continued expressing FGFR1 and FGF9, genes linked to aggressive prostate cancer and bone metastasis [150]. Shokoohmand et al. engineered a 3D model using human osteoblast-derived tissue-engineered constructs with PDX cells on polycaprolactone scaffolds coated with calcium phosphate. The model demonstrated osteomimicry in prostate cancer cells and dynamic interactions between cancer cells and osteoblasts, suggesting its potential as a patient-specific diagnostic platform [156]. These studies highlight the importance of constructing biomimetic environments by integrating PDX and advanced 3D *in vitro* models in sustaining and studying bone-metastatic prostate cancer cells.

### 8.5 Personalized medicine

**Fig. 7:**
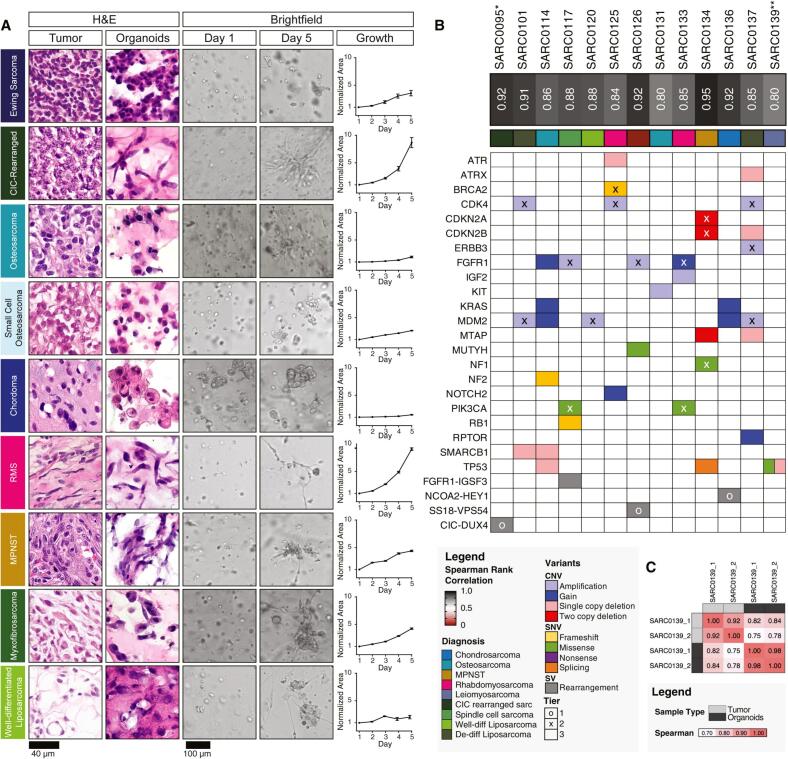
Culture-expandable organoids mirroring parental tumor morphology. (A) Representative images of sarcoma tissues and matched organoids. Columns 1 and 2 show H&E-stained sections of patient-derived sarcomas and their corresponding organoids, respectively. Columns 3 and 4 display representative bright-field images of the same sarcoma-derived organoids in culture on day 1 and day 5. Organoid growth was quantified over time using a machine learning-based image analysis pipeline that segmented in-focus organoids in bright-field images. Growth was expressed as the fold change in cross-sectional area relative to day 1. Data are presented as mean ± SEM. Scale bars: 40 µm (H&E images), 100 µm (bright-field images). (B) Genomic and transcriptomic profiling of selected sarcoma samples and corresponding organoids. Top panel: Spearman’s rank correlation coefficients (dark boxes) indicate transcriptomic similarity between RNA-sequenced tumor samples and their matched organoids, excluding genes with expression below 0.1 transcripts per million in either sample. Bottom panel: Genomic alterations identified using the Dana-Farber Cancer Institute OncoPanel v3.1. Heatmap colors denote the type of alteration: copy number variants (CNVs), single-nucleotide variants (SNVs), or structural variants (SVs). Overlay symbols represent clinical relevance tiers: circles (Tier 1) indicate variants with well-established diagnostic or prognostic value; crosses (Tier 2) indicate variants with potential clinical utility; absence of symbols denotes Tier 3 variants of uncertain significance. (C) Spearman’s rank correlation coefficients comparing transcriptomic profiles of tumor tissue and matched organoids. Reproduced under terms of the CC BY-NC 4.0 license [247]. Copyright 2024, Cell Press.

Personalized medicine is revolutionizing healthcare by tailoring treatments to individual patients based on their unique biological characteristics. In bone tumor research, patient-derived tumor cells in 3D *in vitro* models exemplify this approach, preserving the genetic and phenotypic traits of the original tumors. This enhances translational studies, particularly for rare and heterogeneous tumors like sarcomas, advancing targeted therapies and personalized oncology [246]. A landmark 2024 study utilized patient-derived tumor organoids (PDTOs) from 194 sarcoma specimens spanning 24 subtypes to perform high-throughput drug screening, identifying effective therapies in 59 % of cases. Importantly, organoid responses closely mirrored patient outcomes, demonstrating the value of PDTOs in guiding individualized treatment decisions. Consistently, PDTOs from different sarcoma subtypes maintained the essential histological and molecular characteristics of their source tumors, demonstrating that PDTOs effectively capture both the subtype-specific and individual patient-specific traits of sarcomas (Fig. 7) [247]. Complementing this, the FORTRESS working group recently published detailed guidelines for constructing and characterizing 3D sarcoma tumoroid models to improve reproducibility and standardization in preclinical research [248]. Further strengthening this field, integrative multi-omics approaches with 3D models have revealed novel insights into tumor behavior and drug resistance, paving the way for more refined therapeutic targeting [42]. Additionally, advanced osteosarcoma models now incorporate cancer stem cells and biomimetic scaffolds, enabling more accurate recapitulation of the tumor microenvironment and enhanced drug evaluation [249]. These studies collectively demonstrate the growing utility of PDTOs and 3D *in vitro* models in sarcoma research, paving the way for more personalized and effective therapeutic approaches.

## 9 Clinical relevance of tumor models

The clinical relevance of tumor models is summarized in Table 3. Mouse clinical trials (MCTs) and co-clinical trials, where PDXs are treated in parallel with corresponding human patients, are emerging as powerful tools in precision oncology, particularly for rare and complex tumors like osteosarcoma [250,251]. These models, often referred to as “avatars,” can mirror individual patient responses and predict resistance or efficacy of both first- and second-line therapies [252,253]. MCTs offer several advantages over traditional clinical trials, including the ability to test multiple treatments simultaneously on genetically identical PDX-bearing mice, flexible statistical power through adjustable group sizes, and frequent tumor monitoring. Clinical trials, such as NCT03358628 and NCT04417062, are incorporating osteosarcoma PDX models for drug response profiling and genomic-guided treatment planning. PDTOs have also demonstrated strong predictive capacity in matched therapy cancer studies, including bone [247,254]. The integration of PDTOs into co-clinical trials is advancing precision oncology by enabling personalized treatment strategies. For instance, the ongoing clinical trial NCT06064682 focuses on osteosarcoma, aiming to establish organoid models from patient tumor samples to assess drug responses and inform individualized therapies. Similarly, a broader search on ClinicalTrials.gov reveals multiple studies employing organoids across various malignancies (NCT04859166, NCT05267912, and NCT05932836).

**Table 3:** Clinical relevance of tumor models.

**Model Type**	**Description**	**Clinical use status**	**Applications**	**Refs**
PDXs	Human tumor tissues implanted in immunodeficient mice.	Established	Drug efficacy studies, resistance modeling, co-clinical trials.	[[259], [260], [261], [262]]
PDTOs	3D cultures derived from patient tumor tissue, preserving tumor heterogeneity.	Emerging	Drug sensitivity testing, precision oncology, functional biomarker validation.	[254,263,264]
Engineered 3D *in vitro* models	Scaffold-based or bioprinted 3D models of tumors.	Exploratory	Structural tumor modeling, therapy testing, immune infiltration studies.	[78,265,266]
Tumor-on-chip	Microfluidic platforms replicating dynamic TME components.	Preclinical	Microenvironmental modeling, drug screening under flow and gradient conditions.	[68,267,268]
*Ex vivo* tumor slices	Thin sections of resected tumors cultured short-term.	Preclinical	Short-term drug response, spatial imaging, functional validation.	[257,258]

**Abbreviations**.

PDX; patient-derived xenograft, PDTO; patient-derived tumor organoid, TME; tumor microenvironment, 3D; three-dimensional, ECM; extracellular matrix.

Additionally, engineered 3D *in vitro* models, including scaffold-based and bioprinted constructs, facilitate the study of spatially organized tumor-immune interactions and therapeutic testing [255]. Recent clinical trials are examining the potential of 3D bioprinted tumor constructs in evaluating the efficacy of chemotherapeutic agents, thereby informing clinical decision-making, for example, in gastric (NCT06792149) and pancreatic (NCT05955092) malignancies. Other innovative platforms, such as tumor-on-chip systems, can enable real-time monitoring of tumor behavior under physiologically relevant conditions [256]. *Ex vivo* tumor slices also preserve native tissue architecture and cellular heterogeneity, offering valuable insights into short-term drug responses and spatial drug distribution in human tumors [257,258]. However, these approaches have not considerably integrated into cancer co-clinical trials.

## Conclusion

**Fig. 8:**
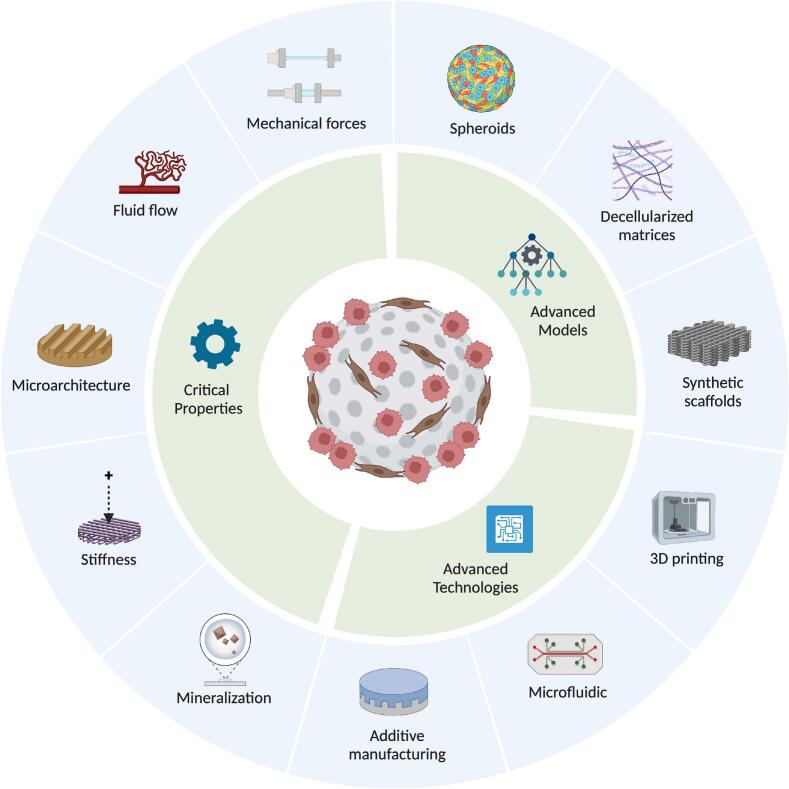
Critical properties as well as advanced methods and technologies for 3D *in vitro* bone tumor models. Created by BioRender.com.

In summary, the development of 3D *in vitro* bone tumor models has made significant strides in bridging the gap between traditional 2D cultures and *in vivo* studies. These models provide a more physiologically relevant environment by incorporating key cellular, structural, and stromal components, mimicking the intricate interactions within the bone microenvironment (Fig. 8). Advances in additive manufacturing, biomaterials, and microfluidic systems have further enhanced their sophistication, improving their ability to replicate tumor dynamics and drug responses. Despite the rapid advancement of 3D tumor models and scaffold-based constructs in recapitulating aspects of tumor biology, several unresolved challenges continue to hinder their full translational potential. Many current models continue to rely on immortalized cancer cell lines, which offer experimental stability and ease of use but exhibit substantial deviations from primary patient tumors at the genomic and proteomic levels. Cancer cells dynamically regulate and remodel the ECM, influencing tumor architecture, signaling, and therapeutic response [269]. On the other hand, incorporating patient-derived cells could help capture patient-specific tumor behavior and enable personalized drug screening; however, issues such as limited tissue availability, low cell yields, and reduced *ex vivo* viability continue to restrict their broader use [13,270,271].

Compounding these biological limitations are technical and standardization-related concerns. Significant variability in matrix composition, culture conditions, and scaffold fabrication methods leads to inconsistent biological outcomes and hampers reproducibility across laboratories [255,272]. Matrigel, a widely used ECM mimic, suffers from ill-defined and batch-dependent composition, which affects cell morphology, growth, and therapeutic response, complicating inter-study comparisons [273]. Scaffold-based models can provide structural biomimicry; however, their reliance on specialized equipment and fabrication processes, e.g., electrospinning and bioprinting, poses accessibility and reproducibility challenges. Furthermore, many current 3D systems fail to fully replicate critical TME features, such as vascularization, immune cell presence, and dynamic nutrient gradients, components that are essential for accurately modeling drug diffusion, metabolism, and resistance [[274], [275], [276]]. Perhaps most critically, the lack of rigorous clinical benchmarking sets a fundamental limitation. Unlike PDXs, which have been extensively correlated with patient outcomes, most engineered 3D models have yet to be systematically validated in predictive clinical workflows. Together, these challenges underscore the urgent need for harmonized protocols, improved material and biological consistency, and robust clinical validation. Addressing these gaps will be essential not only for ensuring reproducibility and reliability but also for accelerating the integration of 3D *in vitro* tumor models into clinically relevant drug development pipelines. With continued innovation, 3D *in vitro* bone tumor models have the potential to become indispensable tools for preclinical drug testing and personalized medicine.

## CRediT authorship contribution statement

**Nicolas Cristini:** Writing – original draft, Investigation. **Mohamadreza Tavakoli:** Writing – original draft, Investigation. **Mehdi Sanati:** Writing – review & editing, Conceptualization. **Saber Amin Yavari:** Writing – review & editing, Supervision, Conceptualization.

## Declaration of competing interest

The authors declare that they have no known competing financial interests or personal relationships that could have appeared to influence the work reported in this paper.
